# How concerning is a SARS-CoV-2 variant of concern? Computational predictions and the variants labeling system

**DOI:** 10.3389/fcimb.2022.868205

**Published:** 2022-08-10

**Authors:** Dana Ashoor, Maryam Marzouq, Khaled Trabelsi, Sadok Chlif, Nasser Abotalib, Noureddine Ben Khalaf, Ahmed R. Ramadan, M-Dahmani Fathallah

**Affiliations:** ^1^ Department of Life Sciences, Health Biotechnology Program - King Fahad Chair for Health Biotechnology, College of Graduate Studies, Arabian Gulf University, Manama, Bahrain; ^2^ Department of Family and Community Medicine, College of Medicine and Medical Sciences, Arabian Gulf University, Manama, Bahrain

**Keywords:** SARS-CoV-2, variants of concern, variant of interest, variant under monitoring, computational prediction

## Abstract

In this study, we evaluated the use of a predictive computational approach for SARS-CoV-2 genetic variations analysis in improving the current variant labeling system. First, we reviewed the basis of the system developed by the World Health Organization (WHO) for the labeling of SARS-CoV-2 genetic variants and the derivative adapted by the United States Centers for Disease Control and Prevention (CDC). Both labeling systems are based on the virus’ major attributes. However, we found that the labeling criteria of the SARS-CoV-2 variants derived from these attributes are not accurately defined and are used differently by the two agencies. Consequently, discrepancies exist between the labels given by WHO and the CDC to the same variants. Our observations suggest that giving the variant of concern (VOC) label to a new variant is premature and might not be appropriate. Therefore, we used a comparative computational approach to predict the effects of the mutations on the virus structure and functions of five VOCs. By linking these data to the criteria used by WHO/CDC for variant labeling, we ascertained that a predictive computational comparative approach of the genetic variations is a good way for rapid and more accurate labeling of SARS-CoV-2 variants. We propose to label all emergent variants, variant under monitoring or variant being monitored (VUM/VBM), and to carry out computational predictive studies with thorough comparison to existing variants, upon which more appropriate and informative labels can be attributed. Furthermore, harmonization of the variant labeling system would be globally beneficial to communicate about and fight the COVID-19 pandemic.

## 1 Introduction

The new coronavirus SARS-CoV-2 that emerged late 2019 is still causing a pandemic of severe acute respiratory syndrome or COVID-19. The pandemic affected over 500 million people and claimed more than six million lives (Johns-Hopkins-University, 2022), thus posing a difficult challenge to the scientific and healthcare communities throughout the world (Malik et al., 2020; Tutelyan et al., 2020; World-Economic-Forum, 2020). Indeed, SARS-CoV-2 is a positive RNA virus that is constantly evolving through the accumulation of various type of mutations (Zhao et al., 2020; Banoun, 2021; Majumdar and Niyogi, 2021; Singh et al., 2021). Even though the majority of these mutations do not affect the virus infectious properties and have no real impact on the progress of the pandemic (Ashoor et al., 2021), some may enhance specific viral attributes that give the virus a selective advantage (Gobeil et al., 2021). Any new variant endowed with selective advantage(s) would favor the virus persistence and nurture the pandemic. Therefore, watching and predicting how the pandemic evolves and communicate it to the public is of paramount importance. The global surveillance of the pandemic is based on multidisciplinary approaches including epidemiological, genetic, structural, and clinical data (Agency, U.H.S, 2021; Campbell et al., 2021; England, P.H, 2021; France, S.P, 2021). This involves the use of a set of relevant criteria to categorize the variants. Toward this end, all international and national health and sanitary authorities have set various strategies to control the evolution of the SARS-CoV-2 pandemic. On 31 May 2021, the World Health Organization (WHO) announced a labeling system to categorize the variants into different levels of priority to better organize the global monitoring and research, and ultimately organize the “infodemic” and communicate more effectively with the public about the adequate response to the emergence of new variants of SARS-CoV-2 (https://www.who.int/). WHO has first developed a system to facilitate naming SARS-CoV-2 variants in addition to the existing nomenclature systems for naming and tracking SARS-CoV-2 genetic lineages established by GISAID (https://www.gisaid.org/), Nextstrain (https://nextstrain.org/), and Pango (https://cov-lineages.org/). These nomenclatures are mostly used by the scientific research community. For practical reasons (particularly to ease the communication), WHO has settled to name the SARS-CoV-2 emerging variants using the Greek letters alphabet sequence (α, β, γ, δ…). Since the SARS-CoV-2 virus is showing high genetic variability (Toyoshima et al., 2020; Yazdani et al., 2021; Dubey et al., 2022), WHO has established a labeling system for the variants into variant of concern (VOC), variant of interest (VOI), and variant under monitoring (VUM). This labeling system is based on definitions related to variant phenotypic attributes such as transmissibility, disease presentation, effect on current diagnostic tests, and response to available vaccines. This system was set to prompt and harmonize the actions needed to control the spread of a given variant. While the variants that emerged sequentially were named α, β, γ, and δ, the last emerging one was named *omicron*. Of interest is that the United States Centers for Disease Control and Prevention (CDC) has also adopted this system of variant classification and labeling but added more labels and labeling criteria and different labels’ change policy (https://www.cdc.gov/). Indeed, while keeping the labels, VOC, VOI, and VUM (calling the later “VBM” for Variant Being Monitored). In addition, the CDC uses an extra label which is “Variant Of High Consequences” (VOHC). As a result, some of the currently known variants are given different labels by each agency. Indeed, according to the CDC, there is no SARS-CoV-2 variant labeled VOI as of December 1, 2021. Furthermore, variants α, β, and γ that are currently labeled VOC by WHO have been deescalated to the VBM label by the CDC as of September 2021 (https://www.cdc.gov/). These discrepancies reflect different views on the labeling of SARS-CoV-2 variants and consequently the use of the labels to set public health actions. On the other hand, in both systems, variant labels can change with more data accumulating for a particular variant.

In this work, we have undertaken an evaluation of the system developed by WHO and adapted by the CDC to label the SARS-CoV-2 variants. We carried out a review of the classification criteria and analyzed how WHO and the CDC use these criteria to label the SARS-CoV-2 variants. Then, we used a comparative computational predictive approach to study the S protein mutations that characterize the five SARS-CoV-2 VOCs. We concluded that computational predictions provide a good ground of evidence for a rapid and more accurate labeling system.

## 2 Materials and methods

### 2.1 Data mining and information sources

We retrieved the genetic, epidemiological, and clinical data on the variants available as of 15 December 2021 from primary and secondary sources including the GISAID (https://www.gisaid.org/) and the Variants (http://covariants.org) data banks. We collected the information on variants naming and labeling from the following sources: WHO: https://www.who.int/en/activities/tracking-SARS-CoV-2-variants/, CDC: https://www.cdc.gov/coronavirus/2019-ncov/variants/variant-info.html#anchor_1632154493691, and PANGO lineages.

### 2.2 Variant sequence retrieval and solvent exposure analysis

SARS-CoV-2 spike protein extracellular domain amino acid sequence was obtained from the National Center for Biological Information (NCBI) protein ID: YP_009724390.1 (NCBI: https://www.ncbi.nlm.nih.gov). Variant-specific mutations were introduced to the collected sequence based on the list published at https://covariants.org/. The sequences corresponding to the different variants (*alpha, beta, gamma, delta*, and *omicron*) were analyzed for solvent exposure and possible epitope residues using the Sequential B-Cell Epitope Predictor server (BepiPred-2.0 server: https://services.healthtech.dtu.dk/service.php?BepiPred-2.0). BepiPred-2.0 is based on a random forest algorithm trained on epitopes annotated from antibody–antigen protein structures (Jespersen et al., 2017).

### 2.3 SARS CoV-2 spike protein furin cut site loop modeling

For the loop modeling, the Phyre2 web server (http://www.sbg.bio.ic.ac.uk/phyre2/html/page.cgi?id=index) (Kelley et al., 2015) was used to generate *alpha, beta, gamma, delta*, and *omicron* variant 3D models of the extracellular spike monomers, and the results were saved and visualized on PyMOL (DeLano, 2002).

### 2.4 Model quality assessment

As a quality assessment for the generated models, the crystallized model of the spike protein (PDB ID 6VXX 2.80A° version 2.4) was downloaded from the RCSB database (https://www.rcsb.org). The structure was cleaned of water and heteroatoms, the complex was split, and a PBD file for a monomer chain was created (chain B) and saved using PyMOL software. This monomer chain B was used as a reference model for the sequence generated models. To define the common contact map between the crystal structures and the generated models, CMView (Vehlow et al., 2011) was used with the following parameters: contact type, Ca; distance cutoff, 8.0; and Needleman-Wunsch alignment. Different contact maps were established between the crystal structure and the models, and the common contact percentage was calculated. Higher common contacts indicate more structural similarity and hence the models are suitable for further analysis. In addition, Tm align (https://zhanggroup.org/TM-align/) (Zhang and Skolnick, 2005) was used to calculate TM score value for each model. The superimposition root mean square deviation (RMSD) was calculated using PyMOL. Low RMSD and TM scoring between 0.5 and 1.0 indicate that the two compared structures (the crystal and the model) has about the same fold.

### 2.5 Mutational analysis: the effect of mutation on the interaction with ACE2 receptor

To analyze the effect of different RBD mutations on different SARS-CoV-2 variant interactions with ACE2 receptor, the PDB ID 6LZG structure was used as a model. The effect of single and accumulated mutations were evaluated by calculating changes in binding affinity (ΔΔG) upon single or multiple mutations using the MutaBind2 server (https://lilab.jysw.suda.edu.cn/research/mutabind2/) (Zhang et al., 2020). In addition, the server provided a structural model that was used to analyze polar interactions by the LigPlot+ software (Laskowski and Swindells, 2011).

## 3 Results

### 3.1 Study of SARS-CoV-2 variant labeling system

**Figure 1:**
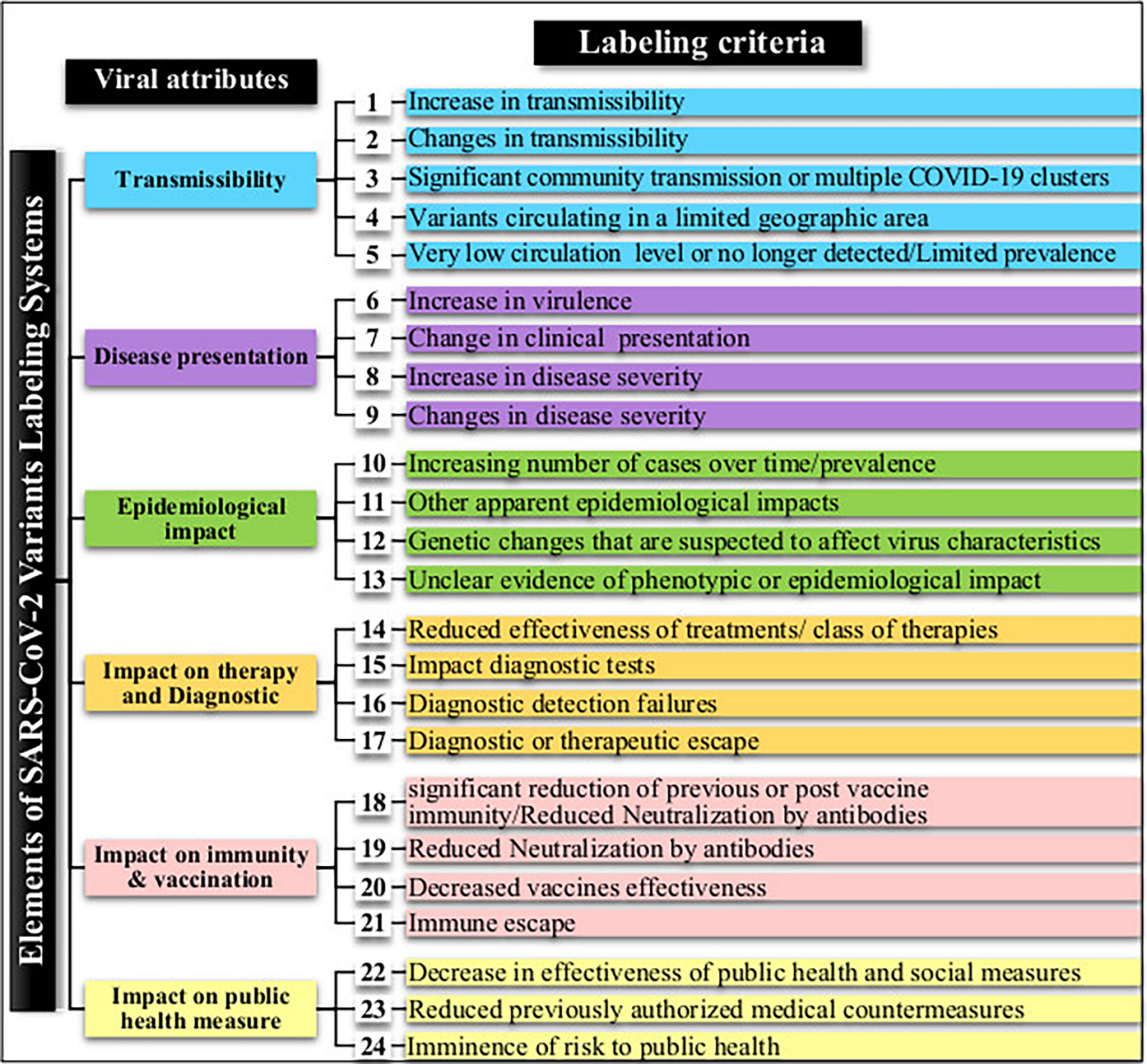
SARS-CoV-2 variant attributes and derived criteria used by the World Health Organization (WHO) and the Centers for Disease Control and Prevention (CDC) for the labeling of emergent variants.

**Figure 2:**
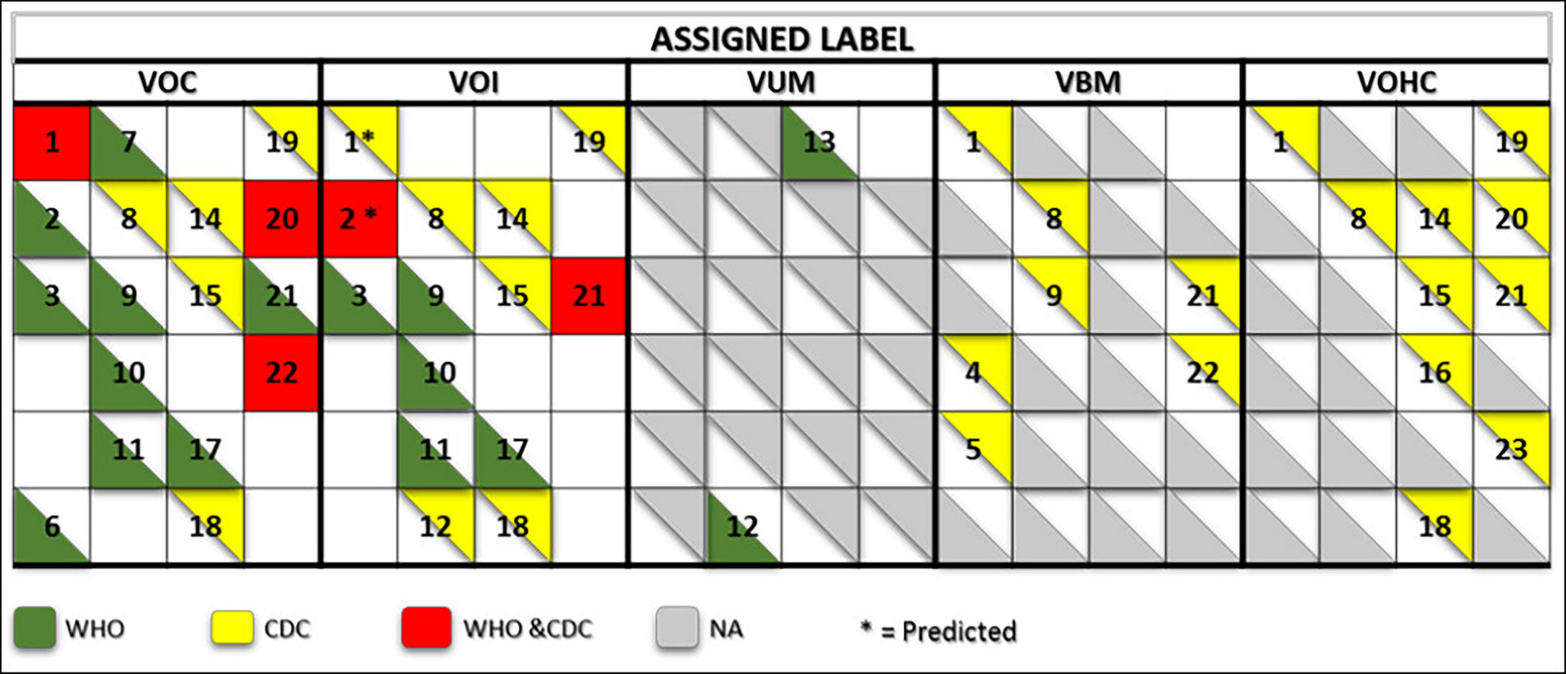
Matrix showing the commonalities and discrepancies of the criteria used by WHO and the CDC to label SARS CoV-2 variants. The green and yellow triangles indicate the criteria used by WHO and the CDC, respectively. The red squares indicate the criteria used by both agencies for a same label. The numbers correspond to the labeling criteria displayed in **Table 1**.

Expert groups at WHO and the CDC use similar labeling systems to classify the new emergent SARS-CoV-2 variants. We retrieved a total of 24 different criteria from the working definitions elaborated by WHO and the CDC to give a particular label to a new variant. These criteria are derived from six viral attributes (**Figure 1**). Each attribute corresponds to a set of criteria that are formulated differently by each agency that considers different criteria in their working definition of each label. The two agencies use common (VOC and VOI) and different (VUM, VBM, and VOHC) labels. Each agency uses generally different combinations of criteria to give a variant a specific label except in five instances shown in **Figure 2** where they use overlapping criteria for the same label (VOC and VOI). Consequently, there are discrepancies in the labels of currently active variants. Indeed, while WHO is currently labeling variants α, β ,γ, δ, and *omicron* as VOC, for the CDC, only variant *omicron* is labeled VOC. Indeed, the CDC declassified variants α, β ,γ, and δ to VBM. In addition, there are no variants currently labeled VOI by the CDC. **Figure 2** illustrates the differential use by WHO and the CDC of the viral attribute-derived criteria to label the SARS-CoV-2 genetic variants.

### 3.2 Attributes of the SARS-CoV-2 major variants of concern

#### 3.2.1 Epidemiological impact and transmissibility

While epidemiological data are available for variants α, β, γ, and δ, data on the *omicron* variant are limited and incomplete. This is mainly because not enough time has elapsed since the emergence of this variant to allow enough data accumulation and meaningful analysis when the variant was given the VOC label. **Table 1** summarizes the initial pieces of data available as of 25 December 2021 on the *omicron* variant. For this variant, the early estimation of transmissibility increase is in the range of three to six. However, the previous data observed with the transmissibility of variant *delta* that became dominant worldwide and the currently observed high spreading/incidence of the *omicron* variant make the prediction of its transmissibility a 100-fold higher than the *delta* variant plausible (Rao and Singh, 2021).

**Table 1:** Global spreading of SARS-CoV-2 *omicron* variant (B.1.1.529).

Date	Transmissibility	Incidence	Country	Reference
9–14 November, Detection of (B.1.1.529) variant	–	70	First 3 Botswana Hong Kong South Africa	Rao and Singh, 2021 https://doi.org/10.47488/dhrp.v1iS5.35 submission of the genomic sequence to GitHub
26 November Classification as a variant of concern	High to very high		8	https://www.who.int/news/item/26-11-2021-classification-of-omicron-(b.1.1.529)-sars-cov-2-variant-of-concern
27 November	Prediction: 100-fold higher than the *Delta* variant	113	8	Rao and Singh, 2021 https://doi.org/10.47488/dhrp.v1iS5.35 https://bnonews.com/index.php/2021/11/omicron-tracker/
2 December	–	390	31	https://www.ecdc.europa.eu/en/news-events/epidemiological-update-omicron-variant-concern-voc-data-2-december-2021
3 December	Three times higher than other variants	486	38	https://www.nature.com/articles/d41586-021-03614-z https://www.ecdc.europa.eu/en/news-events/epidemiological-update-omicron-data-3-december-2021
4 December	–	689	42	https://www.gisaid.org/hcov19-variants/
7 December	Three to six times higher than *Delta*	959	42	https://www.nature.com/articles/d41586-021-03614-z https://www.gisaid.org/hcov19-variants/
21 December	Six times	17,514	78	https://www.gisaid.org/hcov19-variants/
22 December	Six times	20,322	79*	https://www.gisaid.org/hcov19-variants/

*Currently, the omicron has gained global presence, and other subvariants have emerged.

#### 3.2.2 Disease presentation and impact on therapy and diagnostic

COVID-19 has three clinical presentation forms: mild, moderate, and severe. WHO and the CDC use four labeling criteria related to clinical presentation and four others pertaining to the impact of a given variant on therapy and diagnostics. The two agencies use different formulation for all of these criteria (**Figure 1**) and use different combinations of these criteria to label SARS-CoV-2 variants (see **Figure 2**). There is no explicit mention to the disease forms in the variant labeling criteria related to disease presentation in both WHO and CDC variant-labeling usage.

#### 3.2.3 Impact on immunity, vaccination, and public health measures

WHO and the CDC also use different formulations for the criteria related to these attributes and use them differently to attribute a specific label (**Figures 1**, **2**). For instance, for WHO, immune escape is a criterion used to label a variant VOI and VOC. However, the CDC uses it for labeling a variant VOI, VBM, and VOHC. *Omicron* has been shown to have extensively but incompletely evaded the Pfizer BNT162b2 vaccine (Cele et al., 2022), thus fulfilling the criterion of decreased vaccine effectiveness. For the criterion related to the evaluation of an imminent risk to public health a variant can pose, only the CDC uses it for the VOHC label. **Table 2** shows how the five major SARS-CoV-2 variants fulfill the criteria used by WHO and the CDC for variant labeling regardless of the actual labels given by each agency.

**Table 2:** Actual application of the criteria formulated by WHO and the CDC to the five major SARS-CoV-2 variants.

Labeling Criteria																									
Variants
		**1**	**2**	**3**	**4**	**5**	**6**	**7**	**8**	**9**	**10**	**11**	**12**	**13**	**14**	**15**	**16**	**17**	**18**	**19**	**20**	**21**	**22**	**23**	**24**
	**α**																								
	**β**																								
	**γ**																								
	**δ**																								
	**O**							#							#				#			#		#	

### 3.3 Computational approach for the prediction of SARS-CoV-2 viral attributes

**Figure 3:**
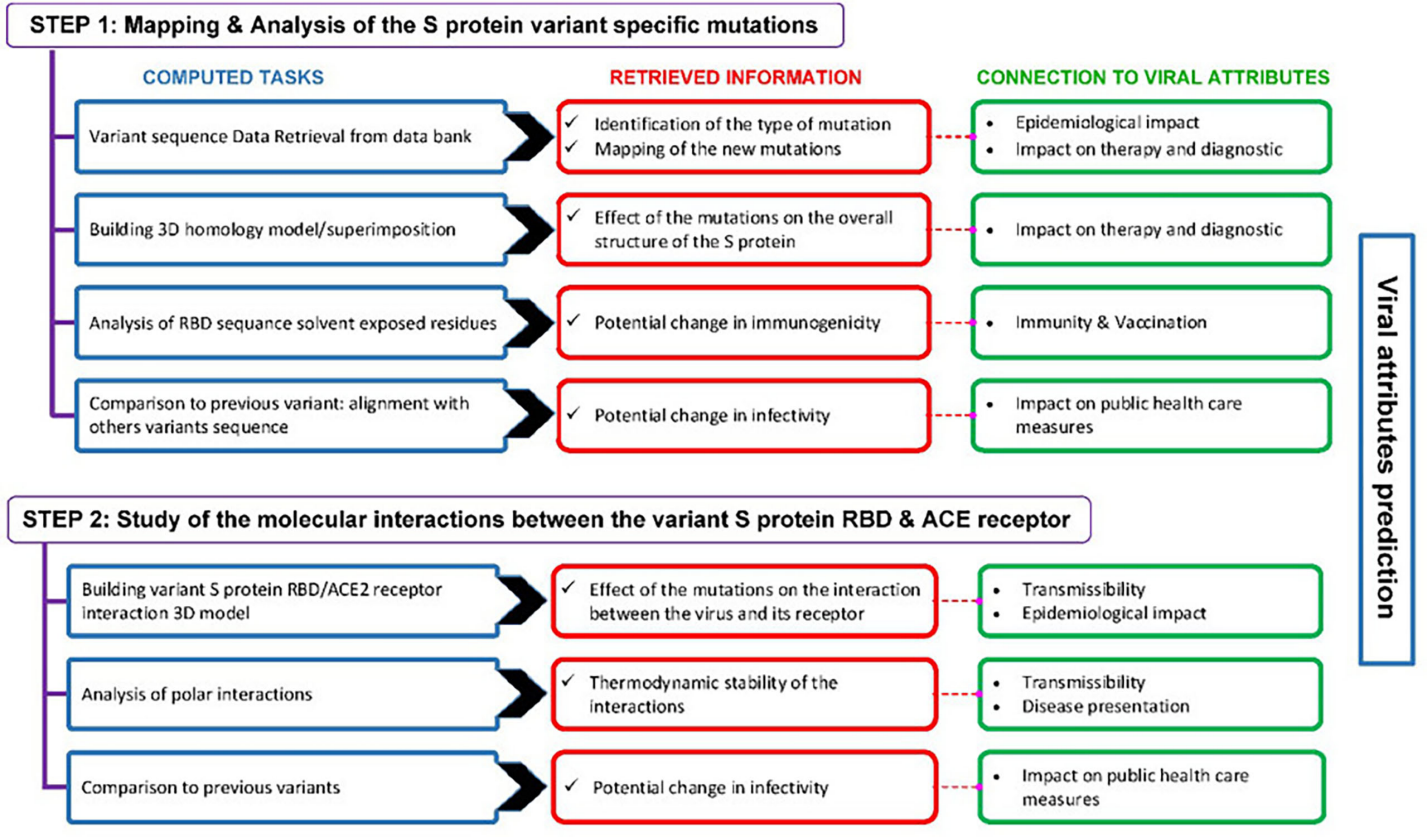
Outline of the computational approach used to predict the viral attributes of SARS-CoV-2 emerging variants. This two-step approach includes a number of computed tasks from which various predictions are retrieved. The predictions are linked to the viral attributes and the criteria that apply are used to label a given variant.

The computational approach we used for the prediction of SARS-CoV-2 emerging variants viral attributes is described in **Figure 3**.

#### 3.3.1 Mutation profile analysis

**Figure 4:**
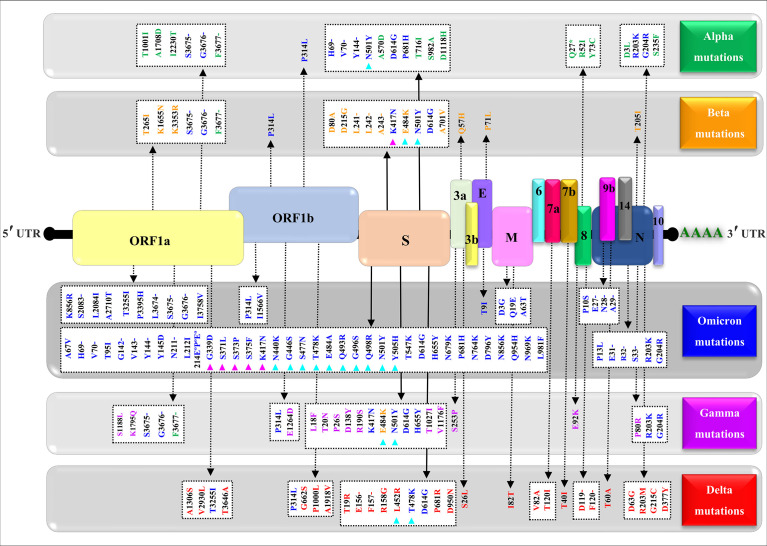
Mapping of the nonsynonymous mutations’ characteristics of the SARS-CoV-2 four variants. A typical genomic organization of SARS-CoV-2 contains the following: 5’ end UTR; open reading frames: ORF 1a and ORF 1b; the structural genes coding for the Spike (S) protein, the Envelope (E), the Membrane (M), and the Nucleocapsid. The accessory genes such as 3a, 3b, 6, 7a, 7b, 8, 10, and 14 are distributed among the structural genes. The 3’ end UTR follows the poly (A) tail. The green, yellow, blue, purple, and red show, respectively, the synonymous mutations characteristic of the alpha (α) variant (B.1.1.7), beta (β) variant (B.1.351), omicron (o) variant (B.1.1.529), gamma (γ) variant (P.1), and delta (δ) variant (B.1.617.2). The (-) represents the deletion, (°) represents the insertion, (*) represents the stop codon, magenta triangles indicate variations in the receptor-binding domain (RBD), and cyan triangles denote variations in the receptor-binding motif (RBM). The NCBI reference sequence for the surface glycoprotein of SARS-CoV-2 is YP_009724390.1.

**Figure 5:**
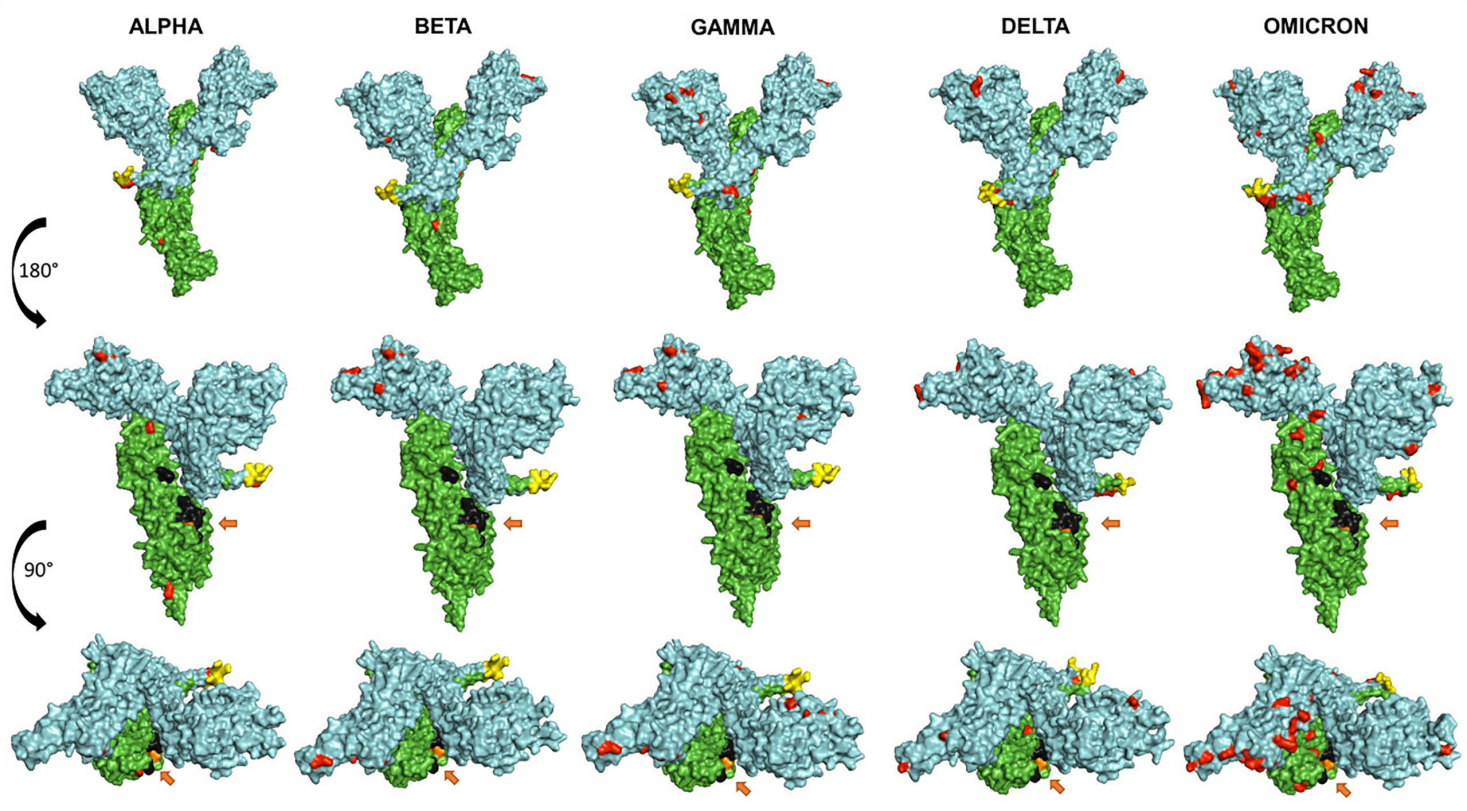
Representation of the surface models of SARS CoV-2 variant S spike protein (monomer). Side views (upper and middle rows) and top view (third row). Color coding is as follows: mutations (red), S1 subunit (cyan), S2 subunit (green), furin cleavage site (yellow), fusion peptides FP1 and FP2 (black), and the arrows show the TMPRSS2 cleavage site (orange).

**Figure 4** shows a comparative mapping of the mutation profile of the *omicron* variant with those of the *alpha, beta, gamma*, and *delta* variants. Most of the mutations affect the SARS-CoV-2 S protein. *Omicron* displays 63 different mutations as compared to the Wuhan strain. Thirty-six mutations occurred in the S spike protein and 15 are clustered in the RBD region. The representation of the mutations in the S protein 3D models of SARS-CoV-2 five VOCs shows that most of the mutations map to the solvent-exposed regions (**Figure 5** and **Supplementary Table 1**).

#### 3.3.2 Effect of omicron S protein mutations on the immunogenicity

**Figure 6:**
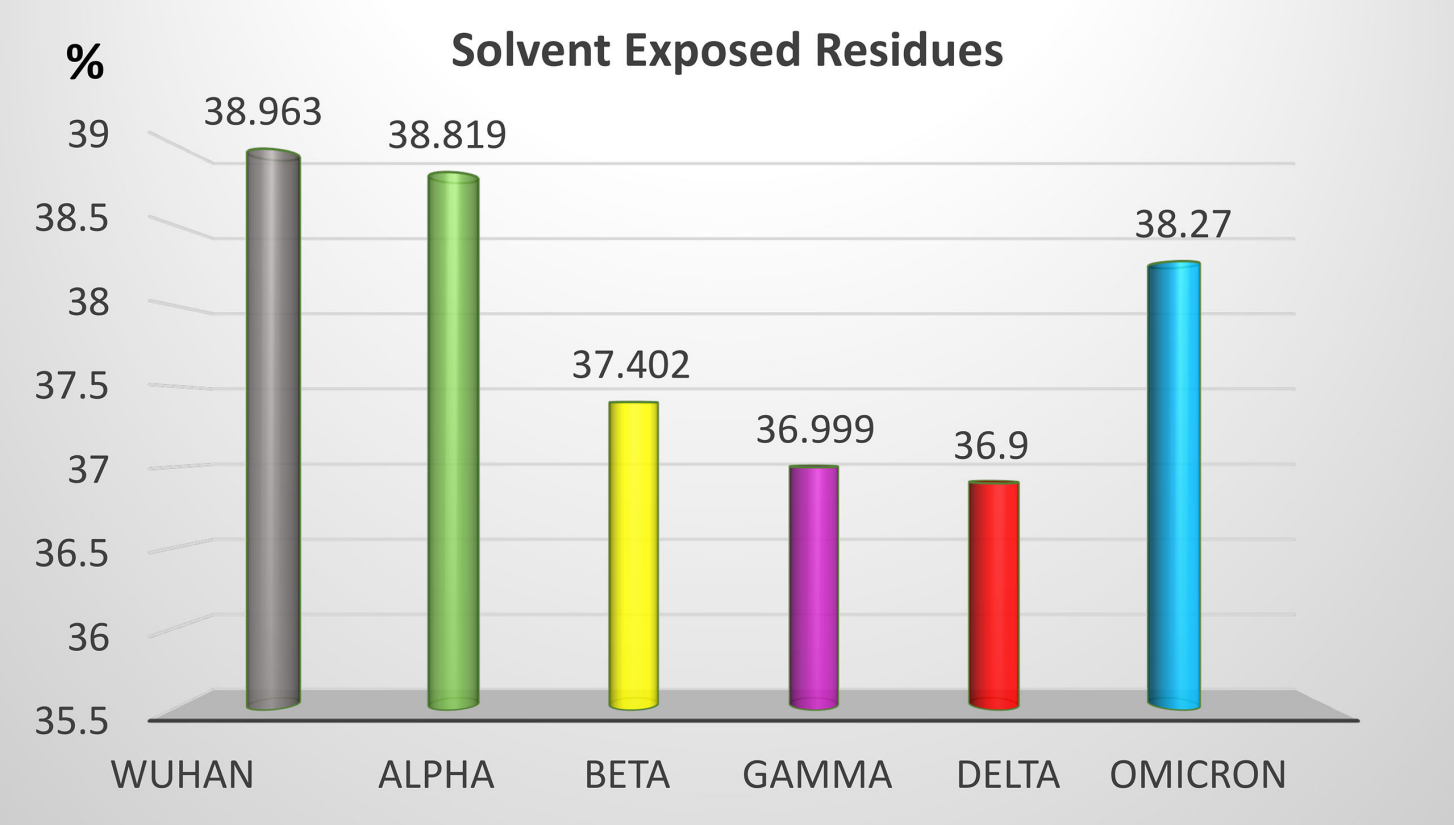
Percentage of solvent-exposed residues in the S protein of the five SARS-CoV-2 VOCs. Solvent-exposed residues at the surface of the S protein are potential epitopes. Variation of the percentage of solvent-exposed residues provide a useful hint for the prediction of immunogenicity changes between variants.

Non-synonymous mutations of the different SARS-CoV-2 variants caused changes on the epitope probability and antibody exposure and hence immunogenicity. Most of the epitope changes are noticeable in the S1 domain of the spike protein. **Figure 6** shows the percentage of the exposed epitope on the different variants. Detailed probability and exposure states of each residue as predicted by BepiPred are listed in **Supplementary Table 1**.

#### 3.3.3 Effect of omicron S protein mutations on the 3D structure of the molecule: Loop modeling and quality assessment

**Figure 7:**
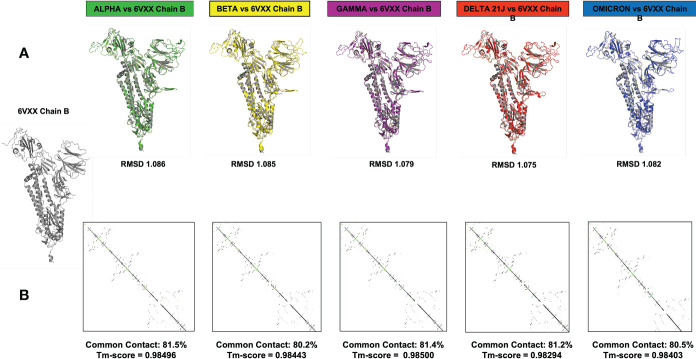
This figure represents the 3D models generated for the α, β, γ, δ, and O variants, their structural analysis, and the quality assessment by superimposition with chain B of the crystallized model PBD 6VXX. **(A)** Superimposition RMSD values. **(B)** Calculated common contact percentages and Tm scores.

Computational methods allow the building of accurate protein models of the SARS-CoV-2 S protein based on data input and the alignment with experimentally solved multiple template molecules. We used the Phyre2 web server to generate 3D models for the extracellular domain of the variants α, β ,γ , δ, and O monomer spike protein (**Figure 7**). The structures were generated with 100% confidence and 84% coverage for *alpha* and *beta*, and 83% coverage for *gamma*, *delta*, and *omicron* by the single highest scoring template. The quality assessment of these models obtained by superimposition with the crystallized structure of the SARS-CoV-2 spike glycoprotein (closed state) 6VXX Chain B showed an RMSD of 1.086 for *alpha*, 1.085 for *beta*, 1.079 for *gamma*, 1.075 for *delta*, and 1.082 for *omicron* (**Figure 5**). The Tm scores were all less than one and both RMSD and TM scoring values are acceptable and indicate high similarity between the crystallized model and the generated one showing the same folds. In addition, the CMView common contact map gave a high similarity score ranging between 80% and 81.5%, keeping in mind that the crystallized structure is missing few residues (gaps) including the furin cleavage loop. What is important here is that these generated models include the furin cleavage site that is lacking in all the crystallized models deposited on the Protein Data Bank (PDB). Moreover, comparison of the contact map percentage and TM-align scoring gave an indication of the similarity between the different models (**Table 3**). Given the highly reliable Tm score and RMSD values, the computationally generated models are suitable to be used for further analysis especially for evaluating the effect of mutations on and around the furin cleavage site.

**Table 3:** Comparison of contact map percentage (red) and TM-align (blue) scoring between the different SARS CoV-2 VOCs. The arrowheads denote the variant used as a reference when calculating the data.

TM-align							
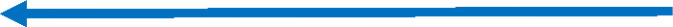							
Contact map %	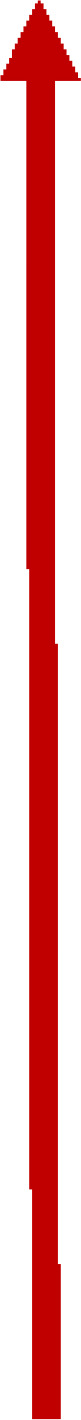		**α**	**β**	**γ**	**δ**	**O**
		**α**		0.99321	0.99811	0.99374	0.99392
		**β**	96.1		0.99361	0.98905	0.99169
		**γ**	98.4	96.9		0.99547	0.99155
		**δ**	95.8	94.8	97.1		0.99041
		**O**	96.1	94.6	96.0	94.6	

#### 3.3.4 The effect of contact residues’ mutations on the SARS-CoV-2 S protein/ACE2 complex thermodynamic stability

Sequence analysis showed that the *omicron* variant has the most mutated RBD with 15 different mutations, out of which, 9 mutations were in the contact residues with ACE2 receptor. The *alpha* variant showed only one mutation in the contact residues (N501Y), *beta* and *gamma* showed three mutations, all of which are in the contact residues, while *delta* showed two mutations, none of which are in the contact residue with ACE2. Analyzing these mutations as a single mutation showed the different effect on the complex of SARS-CoV2 RBD with ACE2 receptor. Where some mutations showed to be deleterious as a single mutation, others have a stabilizing effect. However, the combination of several mutations in the contact residue shows a different effect; in the case of the *omicron* variant, the combination of nine mutations in the contact residues showed to be not deleterious even though they slightly destabilize the complex with ACE2 with an accumulative ΔΔG= 0.18. The *beta* and *gamma* variants with three mutations in the contact residue showed an accumulative ΔΔG of 1.79 and 1.07, respectively, which highly destabilizes the complex and decreases the binding affinity. *Delta* has only two mutations that occur out of the contact residues with a stabilizing effect on the complex and hence increasing affinity to ACE2 and an accumulative ΔΔG = −0.33. The single contact residue mutation of the *alpha* variant (N501Y) showed a destabilizing effect that we reported previously (Ashoor et al., 2021) (**Table 4**).

**Table 4:** Comparison of the mutation profiles of five SARS-CoV-2 VOCs’ S protein in the contact residues with the ACE2 receptor.

6LZGContact residues	*Alpha*B.1.1.7	*Beta*B.1.351	*Gamma*P.1	*Delta*B.1.617.2	*Omicron*BA.1	Single mutations’ ΔΔG	Effect of single mutation
K417		K417N			K417N	0.75	Destabilizing
			K417T			0.18	Destabilizing
G446					G446S	-0.18	Stabilizing
G447							
Y449							
–				L452R		-0.57	Stabilizing
Y453							
L455							
F456							
Y473							
A475							
G476							
S477					S477N	-0.15	Stabilizing
–				T478K		0.19	Destabilizing
E484		E484K	E484K		E484A	0.17	Destabilizing
G485							
F486							
N487							
Y489							
F490							
L492							
Q493					Q493R	0.25	Destabilizing
Y495							
G496					G496S	1.2	Destabilizing
F497							
Q498					Q498R	2.34	Destabilizing
P499							
T500							
N501	N501Y	N501Y	N501Y		N501Y	1.53	Destabilizing
G502							
V503							
Y505					Y505H	1.33	Destabilizing
Q506							
**Accumulative ΔΔG**	1.53	1.79	1.07	-0.33	0.18	NA	NA
**Effect of accumulative mutation**	Destabilizing	Destabilizing	Destabilizing	Stabilizing	Destabilizing	NA	NA

Positive and negative signs correspond respectively to destabilizing (decreases in binding affinity) and stabilizing mutations (increases in binding affinity).

#### 3.3.5 Effect of mutations in the S protein of SARS-CoV-2 major variants on the polar interactions with the ACE2 receptor

Using the crystallized structure PDB ID 6LZG that represents the interaction between SARS-CoV-2 receptor binding domain with ACE2 receptor as a reference, we generated models for the five variants on the LigPlot+ software and analyzed the polar interaction patterns. The interacting residues and type of interactions are shown in **Table 5**. All the variants have one or more mutations on the contact residues except for the *delta* variant. At a first glance to **Table 5** and by comparison, one can spot the interaction pattern similarity between *alpha* and *delta* variants despite the fact that the *alpha* variant showed only one mutation in the contact residue (N501Y) and *delta* has two mutations out of the contact residues (L452R and T478K). Both *alpha* and *delta* variants were reported to be highly transmissible with *delta* being 60% higher (Duong, 2021). *Delta* shows that eight out of nine main polar interactions are present with one missing polar interaction (Spike/ACE2: Asn487/Tyr83) and the addition of new two polar interactions (Gln493/Glu35 and Tyr505/Glu37) and one salt bridge (Glu484/Gln24). Similarly, *alpha* is missing the same polar interactions but shows the same new salt bridge Glu484/Lys31 and no additional new polar interactions. The addition of new polar interactions and the Glu484/Lys31 salt bridge in the *delta* variant could be the cause of the stabilizing effect on the complex with ACE2, which can explain the high transmissibility.

**Table 5:** S protein/ACE2 complex contact residues’ interactions pattern of different SARS-CoV-2 variants.

Spike RBD residue(PDB 6LZG-version 2.4)	Interaction residue on ACE2 isoform 1	SARS CoV-2 variants				
		*Alpha*	*Beta*	*Gamma*	*Delta*	*Omicron*
Lys417	1X Asp30(P) 1X Asp30(H)	1X Asp30(P) 2X Asp30(H)	Missing Missing	Missing Missing	1X Asp30(P) 2X Asp30(H)	Missing Missing
Tyr449	1X Gln42(P) 1X Asp38(P) 3X Asp38(H)	Missing 1X Asp38(P) 3X Asp38(H)	Missing 1X Asp38(P) 3X Asp38(H)	Missing 1X Asp38(P) 3X Asp38(H)	Missing 1X Asp38(P) 3X Asp38(H)	Missing 1X Asp38(P) 3X Asp38(H)
Tyr453	1X His34(H)	1X His34(H)	1X His34(P) 2X His34(H)	1X His34(P) 2X His34(H)	1X His34(H)	1X His34(P) 2X His34(H)
Leu455	1X His34(H)	1X His34(H) 1XAsp30(H)	3X His34(H)	3X His34(H)	1X His34(H) 1XAsp30(H)	3X His34(H)
Phe456	1X Thr27(H) 1X Asp30(H)	1X Thr27(H) 1X Asp30(H)	1X Thr27(H) Missing 1X Lys31(H)	1X Thr27(H) Missing 1X Lys31(H)	1X Thr27(H) 1X Asp30(H)	1X Thr27(H) missing 1X Lys31(H)
Ala475	1X Ser19(P) 2X Ser19(H) 1X Gln24(H)	1X Ser19(P) 2X Ser19(H) 1X Gln24(H) 1X Thr27(H)	1X Ser19(P) 2X Ser19(H) 1X Gln24(H) 1X Thr27(H)	1X Ser19(P) 2X Ser19(H) 1X Gln24(H)	1X Ser19(P) 2X Ser19(H) 1X Gln24(H) 1X Thr27(H)	1X Ser19(P) 2X Ser19(H) 1X Gln24(H) 1X Thr27(H)
Gly476	1X Ser19(H)	1X Ser19(H)	1X Ser19(H)	1X Ser19(H)	1X Ser19(H)	1X Ser19(H)
Glu484		Salt Lys31			Salt Lys31	
Phe486	1X Met82(H) 4X Tyr83(H)	1X Met82(H) 1X Tyr83(H)	1X Met82(H) 1X Tyr83(H)	1X Met82(H) 1X Tyr83(H)	1X Met82(H) 1X Tyr83(H)	1X Met82(H) 1X Tyr83(H)
Asn487	1X Gln24(P) 1X Tyr83(P) 6X Gln24(H) 3X Tyr83(H)	1X Gln24(P) Missing 4X Gln24(H) 2X Tyr83(H)	1X Gln24(P) Missing 4X Gln24(H) 2X Tyr83(H)	1X Gln24(P) Missing 4X Gln24(H) 2X Tyr83(H)	1X Gln24(P) Missing 4X Gln24(H) 2X Tyr83(H)	1X Gln24(P) Missing 4X Gln24(H) 2X Tyr83(H)
Tyr489	1X Thr27(H) 1X Phe28(H)	1X Thr27(H) 1X Phe28(H)	1X Thr27(H) 1X Phe28(H) 1X Lys31(H)	1X Thr27(H) 1X Phe28(H)	1X Thr27(H) 1X Phe28(H)	1X Thr27(H) 1X Phe28(H)
Gln493	1X His34(H) 1X Glu35(H)	1X Glu35(P) 1X His34(H) 2X Glu35(H)	1X Glu35(P) Missing 3X Glu35(H)	1X Glu35(P) Missing 2X Glu35(H)	1X Glu35(P) 1X His34(H) 2X Glu35(H)	1X His34(P) Salt Glu35 4X His34(H) 7X Glu35(H) 1X Asp38(H)
Gly496	1X Lys353(P)	Missing 1X Asp38(H)	Missing 1X Asp38(H)	Missing 1X Asp38(H)	1X Lys353(P) 1X Asp38(H) 3X Lys353(H)	1X Lys353(P) 1X Asp38(P) 5X Asp38(H)
Gln498	1X Gln42(P) 2X Tyr41(H) 2X Gln42(H) 1X Leu45(H)	1X Gln42(P) 6X Tyr41(H) 2X Gln42(H) 1X Leu45(H)	1X Gln42(P) 2X Tyr41(H) 2X Gln42(H) 3X Leu45(H)	1X Gln42(P) 2X Tyr41(H) 2X Gln42(H) 3X Leu45(H)	1X Gln42(P) 4X Tyr41(H) 2X Gln42(H) 1X Leu45(H)	Missing 1X Tyr41(H) 1X Gln42(H) 1X Leu45(H)
Thr500	1X Tyr41(P) 3X Tyr41(H) 1X Asn330(H) 2X Asp355(H) 2X Arg357(H)	1X Tyr41(P) 2X Tyr41(H) 1X Asn330(H) 2X Asp355(H) 2X Arg357(H)	1X Tyr41(P) 2X Tyr41(H) 1X Asn330(H) 1X Asp355(H) 2X Arg357(H)	1X Tyr41(P) 2X Tyr41(H) 1X Asn330(H) 1X Asp355(H) 2X Arg357(H)	1X Tyr41(P) 3X Tyr41(H) 1X Asn330(H) 2X Asp355(H) 2X Arg357(H)	1X Tyr41(P) 2X Tyr41(H) 1X Asn330(H) 2X Asp355(H) 2X Arg357(H)
Asn501	3X Tyr41(H) 1X Lys353(H)	5X Tyr41(H) 5X Lys353(H)	1X Asp38(P) 8X Tyr41(H) 12X Lys353(H)	1X Asp38(P) 9X Tyr41(H) 12X Lys353(H)	4X Tyr41(H) 3X Lys353(H)	1X Lys353(P) 9X Tyr41(H) 16X Lys353(H)
Gly502	1X Lys353(P) 1X Lys353(H) 2X Gly354(H)	1X Lys353(P) 1X Lys353(H) 2X Gly354(H)	1X Lys353(P) 1X Lys353(H) 2X Gly354(H)	1X Lys353(P) 1X Lys353(H) 2X Gly354(H)	1X Lys353(P) 1X Lys353(H) 2X Gly354(H)	1X Lys353(P) 1X Lys353(H) 2X Gly354(H)
Tyr505	5X Lys353(H) 1X Gly354(H)	4X Lys353(H) 1X Gly354(H)	1X Glu37(P) 12X Lys353(H) 1X Gly354(H) 4X Glu37(H)	1X Glu37(P) 11X Lys353(H) 1X Gly354(H) 4X Glu37(H)	1X Glu37(P) 12X Lys353(H) Missing 3X Glu37(H)	4X Lys353(H) 1X Gly354(H)
**Total interactions**	10P/56H	8P/1 salt/63H	10P/83H	10P/80H	10P/1 salt/72H	10P/1 salt/85H
**ΔΔG**		1.53	1.79	1.07	−0.33	0.18
**Effect**		**Destabilizing**	**Destabilizing**	**Destabilizing**	**Stabilizing**	**Destabilizing**

Polar interactions (P), hydrophobic interactions (H), Missing interactions are highlighted in red, new interactions highlighted in blue, salt bridges are highlighted in green. SARS-CoV-2 Wuhan strain sequence was used as reference sequence.

In addition to Glu484, other mutations on RBD binding hot spot residues have been also linked to antibody binding and neutralization including mutations on Lys417, Gly446, Phe456, Asn501, Gly477, and Asp614 (Greaney et al., 2021a; Liu et al., 2021b; Montefiori, 2021; Supasa et al., 2021). Interestingly, the *omicron* variant compiles all these mutations (**Table 5**), indicating a potential higher degree of immune escape. Additionally, *omicron* has a consecutive mutation on residues Gln493, Gly496, and Gln498 that dramatically affect the interaction with ACE2. These residues are structurally in the receptor binding ridge and Gln493 is known to form with Leu455 the two receptor binding motif (RBM) stabilizing hot spots and to be a target for some therapeutic antibodies (Shang et al., 2020; Greaney et al., 2021b). This interaction-disrupted profile may sum up into a less stable complex with ACE2 (ΔΔG = 0.18) (**Table 5**) and loss of antibody neutralization. Furthermore, the comparison of the interactions’ differences between SARS-CoV-2 variants showed some interactions that are conserved in all the variants, suggesting their importance in the complex stability.

## 3 Discussion

Despite significant global healthcare measures and social mitigation efforts along with the availability of a number of vaccines, the SARS-CoV-2, COVID-19 pandemic entered its third year and the numbers of cases are soaring worldwide (https://www.gisaid.org/, https://coronavirus.jhu.edu/map.html). This is mainly due to the emergence of new and more fit viral variants that continue to fuel this pandemic. To create awareness about any additional health issue a new variant may cause, WHO, the world’s most influential health agency, has developed a labeling system to classify the new variants, in addition to the conventional nomenclature. The CDC has adopted this labeling system but introduced major changes. Among the labels used by WHO and the CDC is the label “Variant of Concern or VOC”. The word “concern” is synonymous of anguish, anxiety, and apprehension. It naturally enjoins fear and disturbance, which predispose taking immediate action and making odd changes. Indeed, when a new variant is labeled VOC, countries are inclined to close their borders and take drastic countermeasures. This was particularly striking when a number of countries banned travels from South Africa, which was the first country to describe and report the genomic sequence of the *omicron* variant. While preventive and cautionary actions are mandatory to control pandemics, these should be based on solid scientific evidence. However, it takes time and coordinated efforts for the scientific community to generate the data needed to accurately label new variants according to criteria such as transmissibility, disease severity, and change in the epidemiological pattern, immune escape, and resistance to previously neutralizing antibodies, efficacy of existing therapies, or vaccine efficiency. Meanwhile, the rapid pandemic progress requires timely response including a good communication system. In this study, we highlight some flaws in the labeling system developed by WHO mainly to ease communication with the national health authorities and the public. Indeed, in addition to the non-appropriate wording for labeling emergent variants, the combination of criteria used to define a variant label as shown in WHO and the CDC web sites, is not accurate. Indeed, in their definitions of the different labels, both agencies build a combination of criteria using the prefix “OR” but not “AND”. This introduces a confusion that is amplified by the CDC use of more labels, and different formulation and different combinations of the criteria used to attribute a label to a new variant. According to WHO, a label is supposedly assigned to a variant through a comparative assessment with the previous ones. Even though SARS-CoV-2 variants are primarily detected upon the virus genomic sequence changes and particularly mutations in important functional regions, the genetic variations do not clearly appear among the viral attributes WHO and the CDC use to formulate their definition of the different variants. The labeling system developed by WHO and the definitions of the criteria for each label used by both WHO and the CDC do not mention a comparative assessment of the genetic variations but rather retain clinical, epidemiological transmissibility and other non-genetic viral attributes. Such parameters need a substantial time to be accurately determined. It seems that the current labeling system relies more on fear from emergent genetic variations, “the super killer virus”, than evidence about the impacts of mutations (Caini et al., 2018). Thus, it seems premature and not appropriate to give a new variant the VOC label. Even when a greater transmissibility can be established, this does not necessarily mean greater severity. For example, the very transmissible H1N1 influenza virus variant was not as severe as many other influenza viruses and the epidemic has naturally faded away (Grubaugh et al., 2020). With reference to the WHO variant labeling system, H1N1 could have been labeled VOC while it has never been one. Therefore, to solve the dilemma between the necessity of rapidly labeling a new variant and the lack of scientific evidence, a good approach is to thoroughly analyze the genetic variations observed to rapidly generate the best data on the potential attributes of the new viral variant. To achieve this analysis, the computational prediction of variant effect on protein stability, function, and interaction is a very useful way to determine the variant “importance”. Several methods can be used based on the availability or non-availability of the 3D structure of the protein. In this study, we show that computational prediction and *in silico* comparative studies between new variants and older more characterized ones can provide good insights into the impact of the mutations and thus the potential behavior of the emergent variants. Indeed, the observation that most of the mutations in SARS-CoV-2 VOCs map to the solvent-exposed regions along with the comparison of antigenicity predictions (Crooke et al., 2020; Vashi et al., 2020; Chen et al., 2021) represents a good basis to make a fair assumption on a potential immune escape and/or a likely reduction of antibody neutralization and to anticipate an evaluation of vaccine effectiveness. In addition, the comparative analysis of the S protein 3D structure between the five SARS-CoV-2 VOCs showed an identical overall folding as demonstrated by the RMSD values and Tm scores of each model. Meanwhile, the comparison of contact residue maps gave a fine-tuning of the structural divergence, which suggests potential functional differences that need to be further analyzed.

Furthermore, computational studies of the mutation effect on the thermodynamic stability of the S protein/ACE2 complex and the comparative analysis of the pattern of polar interactions of the different variant with the ACE2 receptor give good indications to predict transmissibility and virulence and draw some plausible epidemiological scenario. Indeed, the effect of mutations can be computed as single or combined mutations. The data we obtained with the combined mutations of the *omicron* variant that have a much higher number of mutations than the other variants show that this variant engages into a more stable interaction with ACE2 than the β and γ variants do and has more interactions than the *delta* variant.

For the other variant labeling criteria, several studies discussed the importance of the Glu484Lys mutation in the interaction with ACE2 and immune escape (Greaney et al., 2021b; Makdasi et al., 2021). This was also observed with the *beta* and *gamma* variant that carry Glu484Lys mutation. It was reported that the *beta* variant has reduced antibody neutralization compared to the *delta* variant. Moreover, *beta*’s resistance to neutralizing antibodies increased by 9.4-fold to convalescent plasma and 10.3- to 12.4-fold for sera from individuals who have been vaccinated (Liu et al., 2021a). In addition, it was suggested that new variants with the same mutation might bear new challenges for current vaccines or monoclonal antibody therapies (Krause et al., 2021; Zhou and Wang, 2021).

Meanwhile, our analysis of the pattern of polar interactions between the different variants shows that *omicron* has an extra 13 hydrogen bonds as compared to variant *delta*. In addition, we noticed the presence in the more transmissible *alpha* and *delta* variants of an extra salt bridge between the S protein Glu 484 and ACE2 lysine 31 and the presence of an extra salt bridge between the S protein Gln 493 and ACE2 Glu 35 in the *omicron* variant. This observation, combined with the observed high number of H bonds and the thermodynamic stability data, predicts a potentially more efficient entry into host cells and enhanced transmissibility of the *omicron* variant, which has been confirmed since the description of the *omicron* mutation profile. Nevertheless, relying on the study of a single mutation or the computing of one biophysical feature of a variant structure to predict how a viral attribute would evolve is not sufficient. Furthermore, multiple genes can control epidemiologically relevant viral attributes such as the mode of transmission and virulence. Therefore, it is recommended to integrate the data on multiple mutations with computation of various viral structural features to make the best predictions and attribute the right label to a given variant.

In conclusion, the system of SARS-CoV-2 labeling developed by WHO and amended by the CDC has some major flaws. Relying on the integrated biophysical and structural data generated from computational comparative predictions of the likely behavior of a new variant would help in the rapid and accurate labeling of emergent variants. Meanwhile, given our provisional and incomplete knowledge and the uncertain nature of the COVID-19 pandemic, it would be wise to operate in epistemic self-abnegation, use the best tools and knowledge we have at hand, and introduce revisions whenever new evidence becomes available.

## Data availability statement

The raw data supporting the conclusions of this article will be made available by the authors, without undue reservation.

## Author contributions

DA: *in silico* analysis, methodology, data curation, writing, and editing. MM: mutations’ review, illustrations, figures, and tables. KT: data retrieval from data banks and formatting. SC: analysis and illustration of the WHO and CDC variant-labeling criteria. NA: data retrieval and iconography. NK: review of the *in silico* study. AR: data cross-checking and coordination. M-DF: project conception, work design, data analysis, writing, editing, and supervision. All authors contributed to the article and approved the submitted version.

## Funding

Grant LS_COVID 19, 2020 from the Arabian Gulf University.

## Conflict of interest

The authors declare that the research was conducted in the absence of any commercial or financial relationships that could be construed as a potential conflict of interest.

## Publisher’s note

All claims expressed in this article are solely those of the authors and do not necessarily represent those of their affiliated organizations, or those of the publisher, the editors and the reviewers. Any product that may be evaluated in this article, or claim that may be made by its manufacturer, is not guaranteed or endorsed by the publisher.
